# *Agathobaculum butyriciproducens* Shows Neuroprotective Effects in a 6-OHDA-Induced Mouse Model of Parkinson&rsquo;s Disease

**DOI:** 10.4014/jmb.2205.05032

**Published:** 2022-09-05

**Authors:** Da Woon Lee, Young-Kyoung Ryu, Dong-Ho Chang, Hye-Yeon Park, Jun Go, So-Young Maeng, Dae Youn Hwang, Byoung-Chan Kim, Chul-Ho Lee, Kyoung-Shim Kim

**Affiliations:** 1 Laboratory Animal Resource Center, Korea Research Institute of Bioscience and Biotechnology (KRIBB), Daejeon 34141, Republic of Korea; 2 Department of Biomaterials Science, College of Natural Resources and Life Science and Industry Convergence Research Institute, Pusan National University, Miryang 50463, Republic of Korea; 3 Microbiome Convergence Research Center, Korea Research Institute of Bioscience and Biotechnology (KRIBB), Daejeon 34141, Republic of Korea; 4 College of Biosciences and Biotechnology, Chung-Nam National University, Daejeon 34134, Republic of Korea; 5 HealthBiome, Inc., Daejeon 34141, Republic of Korea; 6 Department of Biosystems and Bioengineering, University of Science and Technology (UST), Daejeon 34113, Republic of Korea

**Keywords:** SR79, oxidative stress, inflammation, mouse model, Parkinson’s disease

## Abstract

Parkinson’s disease (PD) is the second-most prevalent neurodegenerative disease and is characterized by dopaminergic neuronal death in the midbrain. Recently, the association between alterations in PD pathology and the gut microbiota has been explored. Microbiota-targeted interventions have been suggested as a novel therapeutic approach for PD. *Agathobaculum butyriciproducens* SR79^T^ (SR79) is an anaerobic bacterium. Previously, we showed that SR79 treatment induced cognitive improvement and reduced Alzheimer's disease pathologies in a mouse model. In this study, we hypothesized that SR79 treatment may have beneficial effects on PD pathology. To investigate the therapeutic effects of SR79 on PD, 6-hydroxydopamine (6-OHDA)-induced mouse models were used. D-Amphetamine sulfate (d-AMPH)-induced behavioral rotations and dopaminergic cell death were analyzed in unilateral 6-OHDA-lesioned mice. Treatment with SR79 significantly decreased ipsilateral rotations induced by d-AMPH. Moreover, SR79 treatment markedly activated the AKT/GSK3β signaling pathway in the striatum. In addition, SR79 treatment affected the Nrf2/ARE signaling pathway and its downstream target genes in the striatum of 6-OHDA-lesioned mice. Our findings suggest a protective role of SR79 in 6-OHDA-induced toxicity by regulating the AKT/Nrf2/ARE signaling pathway and astrocyte activation. Thus, SR79 may be a potential microbe-based intervention and therapeutic strategy for PD.

## Introduction

Parkinson’s disease (PD) is a complex neurodegenerative disorder characterized by dopaminergic neurodegeneration and motor impairments such as resting tremors, postural instability, and muscle rigidity [1, 2]. In addition to the dysregulation of movement, non-motor symptoms, including mental illness, cognitive dysfunction, and sensory dysfunction have also been observed in patients with PD [3, 4]. Gastrointestinal (GI) dysfunction in PD has been reported as one of the most common non-motor symptoms [5, 6]. Before motor symptoms, constipation is highly prevalent [7], and the prevalence of GI dysfunction in PD is up to 80% [8].

The microbiota has emerged as a major regulator of gut and brain function [9]. The vagus nerve and enteric nervous system are affected in the early phases of PD [10]. The gut microbiota may influence brain–gut axis interactions. Germ-free animals lacking exposure to microorganisms show alterations in behavioral phenotypes and differences in neurotransmission [11, 12]. The use of fecal microbiota transplants (FMT) or probiotics induces neurobehavioral changes in animals [13, 14] and influences psychological function [15]. Temporary improvement in leg tremors has been observed in an FMT study in PD patients [16]. Several in vivo animal studies have shown improved motor performance and reduced degeneration of midbrain dopaminergic neurons, indicating the neuroprotective effect of probiotics [17-19]. In addition, fewer anti-inflammatory gut bacteria, such as butyric acid-producing bacteria, and more pro-inflammatory gut bacteria, such as Proteobacteria, have been found in patients with PD [20, 21].

*Agathobaculum butyriciproducens* SR79^T^ (SR79) is a gram-positive and strict anaerobic gut bacterium belonging to a new genus within the family *Oscillospiarceae* [22]. A major fermented product of SR79 is butyric acid [22]. Previously, we showed the beneficial effects of SR79 in mouse models of Alzheimer’s disease (AD) [23]. Lipopolysaccharide- and amyloid-β plaque-induced cognitive impairment was improved by the administration of SR79 [23]. Interestingly, Aβ plaque-induced inflammation is markedly reduced by SR79 treatment in APP/PS1 transgenic mice [23]. Chronic neuroinflammation is associated with PD development [24]. Evidence suggests that activation of microglia or astrocytes could contribute to the neuropathology of PD and that downregulation of inflammatory responses may be important in slowing the progression of PD [25-28]. We hypothesized that SR79 may be neuroprotective in PD. In the present study, we examined whether d-amphetamine (d-AMPH)-induced rotation and dopaminergic neuronal death were mitigated by the administration of SR79 in a 6-hydroxydopamine (6-OHDA)-induced rodent model of PD. Molecular biological changes were investigated on both the unlesioned and 6-OHDA-lesioned sides of the striatum. Furthermore, anti-ionized calcium-binding adapter molecule 1 (Iba-1) and glial fibrillary acidic protein (GFAP) immunoreactivity were analyzed in the intact and 6-OHDA-lesioned sides of the substantia nigra.

## Materials and Methods

### Culture and Treatment of SR79

The preparation and culture of *Agathobaculum butyriciproducens* SR79^T^ (SR79, DSM 100391^T^) have been described previously [22, 23]. All procedures were performed under strict anaerobic conditions as previously described [22, 29]. Briefly, the glycerol stock of SR79 was inoculated into a Hungate tube containing anaerobic DSM 104 medium (5 ml, DSMZ, Leibniz Institute, Germany) and incubated at 37°C for 1 day. The DSM 104 medium (1 L) contained trypticase peptone (5 g), peptone (5 g), yeast extract (10 g), beef extract (5 g), glucose (5 g), Tween 80 (1 ml), CaCl_2_ (10 mg), MgSO_4_ (20 mg), K_2_HPO_4_ (2.04 g), KH_2_PO_4_ (40 mg), NaHCO_3_ (400 mg), NaCl (80 mg), haemin solution (10 ml of 0.5 mg/ml), and vitamin K1 solution (0.2 ml of 5 mg/ml). Anaerobic DSM 104 agar plates (1.6%, w/v) were prepared in an anaerobic chamber (Coy Laboratory Products, USA) with N_2_/CO_2_/H_2_ (86:7:7) gas phase for 3 days. The liquid-cultured SR79 was spread on DSM 104 agar plates and incubated at 37°C in an anaerobic chamber for 1 day. The bacteria were collected by scraping with a loop (Thermo Scientific, USA) and resuspended in anaerobic phosphate-buffered saline (PBS). The cultures were washed and concentrated in anaerobic PBS containing 25% (v/v) glycerol to a final concentration of 1.5 × 10^9^ colony-forming units (CFU)/ml under strict anaerobic conditions. The number of viable cells in the PBS suspension was measured by evaluating the CFU on DSM agar plates. The SR79 culture was aliquoted into the proper number of cells in screw-capped tubes with a rubber ring, double-sealed with zipper bags, and stored in a -80°C deep-freezer before use. SR79 was administered to the mice daily via oral gavage at a dose of 2 × 10^8^ CFU/day.

### Animals

Eight-week-old male C57BL/6J mice (B6, 22–28 g) were obtained from the Korea Research Institute of Bioscience and Biotechnology (KRIBB). All mice were maintained under specific-pathogen-free (SPF) conditions. The animals were housed in a humidity- and temperature-controlled environment on a 12 h light/dark cycle with ad libitum access to autoclaved water and gamma-irradiated food (2018s, Envigo Teklad, USA). After one week of acclimatization, B6 mice at nine weeks of age were randomized into the vehicle (*n* = 8) and SR79 (*n* = 8) groups. SR79 and vehicle were administered daily by oral gavage for 7 days, followed by intra-striatal injection with 6-hydroxydopamine (6-OHDA). The vehicle and SR79 were administered for 10 days. All mice were handled in accordance with the Guidelines of Animal Care of KRIBB (KRIBB-AEC-16115), and experiments were performed in accordance with the Guide for the Care and Use of Laboratory Animals published by the United States National Institutes of Health.

### Drugs

The compound 6-OHDA was purchased from Sigma-Aldrich (USA) and was diluted in saline containing 0.02% ascorbic acid. Desipramine hydrochloride was purchased from Sigma-Aldrich and d-AMPH was purchased from United States Pharmacopeia (USA). Desipramine and d-AMPH were dissolved in saline.

### 6-OHDA Lesion

The 6-OHDA lesioning has been described previously [30-32]. Mice were pretreated with desipramine (25 mg/kg, intraperitoneally) 30 min before surgery to prevent noradrenergic neuronal damage. The mice were anesthetized with a mixture of xylazine hydrochloride and ketamine hydrochloride. The mice were mounted in a stereotactic frame (Stoelting Europe, Dublin, Ireland). The mice received injections of 6-OHDA in a volume of 3 μl (2 μg/μl) into the left dorsal striatum at the following coordinates from the mouse brain atlas of Paxinos and Franklin [33]:+1.2 mm anteroposterior and -1.8 mm lateral from the bregma and −3.6 mm dorsoventral from the skull. The mice were kept on a warming plate until they awoke from anesthesia and were subsequently returned to their home cages until use. To avoid dehydration, glucose (JW Pharmaceutical, Korea) was administered to the mice (50 mg/ml, 0.1 ml/body weight (10 g), subcutaneous injection) for 3 days. During the first week after surgery, water-soaked food pellets were placed in a shallow vessel on the floor of the cages in the evening. The striatum-lesioning procedure resulted in a mortality rate of 6.25%. One vehicle-treated mouse died after surgery.

### D-AMPH-Induced Rotation Test

The d-AMPH-induced behavioral rotation test was performed as previously described [32, 34]. The test was performed 7 days after the 6-OHDA injection. After the d-AMPH injection (5 mg/kg, i.p.), turning behavior was recorded for 30 min using a rotation cylinder. A SMART video tracking program (Panlab, Spain) was used for data analysis.

### Immunohistochemistry

Immunohistochemistry was performed as previously described [23, 34]. The brain samples were fixed in 4%paraformaldehyde (Sigma-Aldrich). The fixed brain was cut into 40 μm coronal sections on a vibratome (VT1000S, Leica, Germany). The sections were incubated in PBS containing 3% H_2_O_2_ and washed three times in PBS containing 0.1% Triton X-100. The sections were blocked with serum for 1 h at room temperature and incubated overnight at 4°C with anti-tyrosine hydroxylase (TH; Pel-Freeze, USA), anti-Iba-1 (Wako, Japan), or anti-GFAP (Dako, USA). The sections were incubated in an avidin/biotin ABC complex (ABC Kit, Vector Laboratories) with a biotinylated anti-rabbit IgG secondary antibody (Vector Laboratories, USA). All sections were reacted with 3,3'-diaminobenzidine (Sigma-Aldrich Co.) and mounted on microscope slides. TH-positive cells and Iba-1- and GFAP-positive areas were analyzed using light microscopy (Olympus Corporation, Japan) and the MetaMorph software (Molecular Devices Inc., USA).

### Western Blotting

Western blotting was performed as previously described [23, 35]. Thirty minutes after the 17 day SR79 treatment schedule, the mice were euthanized by quick cervical dislocation, and their brains were removed. Ipsilateral and contralateral striata were homogenized in 1× RIPA buffer (Millipore, Germany) containing a cocktail of protease inhibitors (Roche, Germany). The homogenates were centrifuged at 1,000 ×*g* at 4°C for 10 min, and the supernatants were centrifuged at 17,000 ×*g* at 4°C for 10 min to obtain the supernatant. Protein samples were resolved by SDS-PAGE and then transferred onto a polyvinylidene fluoride membrane (Bio-Rad). Blots were incubated with blocking serum overnight at 4°C with the following primary antibodies: anti-phospho-Akt (Ser473, Cell Signaling Technology (CST), 1:1,000), anti-Akt (CST, 1:1,000), anti-phospho-GSK3β (Ser9, CST, 1:1,000), anti-GSK-3β (CST, 1:1,000), anti-TH (Pel-Freeze, 1:1,000), anti-actin (Millipore, 1:10,000), and anti-α-tubulin (CST, 1:1,000).

### Quantitative Real-Time Polymerase Chain Reaction (RT-PCR)

RT-PCR was performed as described previously [34, 36]. The dissected striatal tissues were homogenized using the TRI reagent (Sigma-Aldrich). The purity and quality of the isolated RNA were determined using a NanoDrop ND-1000 spectrophotometer (Thermo Fisher Scientific, Inc., USA). cDNA synthesis was performed using a Reverse Transcription System (Promega, USA) according to the manufacturer’s instructions. qPCR was performed using an Optical 96-Well StepOnePlus Real-Time PCR System (Applied Biosystems, UK). Comparative qPCR was performed using the SYBR Green Master Mix (Applied Biosystems). The expression levels of the target genes were normalized to the expression of 18s ribosomal RNA (18S rRNA) and quantified using the comparative cycle threshold method (2-ΔΔCt) [33]. The mRNA expression levels of genes, including superoxide dismutase 1 (*Sod1*), superoxide dismutase 2 (*Sod2*), superoxide dismutase 3 (*Sod3*), NAD(P)H quinone dehydrogenase 1 (*Nqo1*), nuclear factor erythroid 2-related factor 2 (*Nrf2*), heme oxygenase 1 (*Ho1*), B-cell leukemia/lymphoma 2 (*Bcl2*), Bcl2-associated X protein (*Bax*), Bcl2-associated agonist of cell death (*Bad*), tyrosine hydroxylase *Th*, and 18S rRNA, were analyzed. Gene names and primer sequences are summarized in Table 1.

### Statistical Analysis

Two-sample comparisons were carried out using Student’s *t*-test, while multiple comparisons were made using one-way ANOVA followed by Tukey’s multiple range test (GraphPad Software, USA). All data are presented as the mean ± standard error of the mean (SEM), and statistical differences were accepted at the 5% level unless otherwise indicated.

## Results

### SR79 Improves Motor Impairment in Unilateral 6-OHDA-Lesioned Mice

**Fig. 1:**
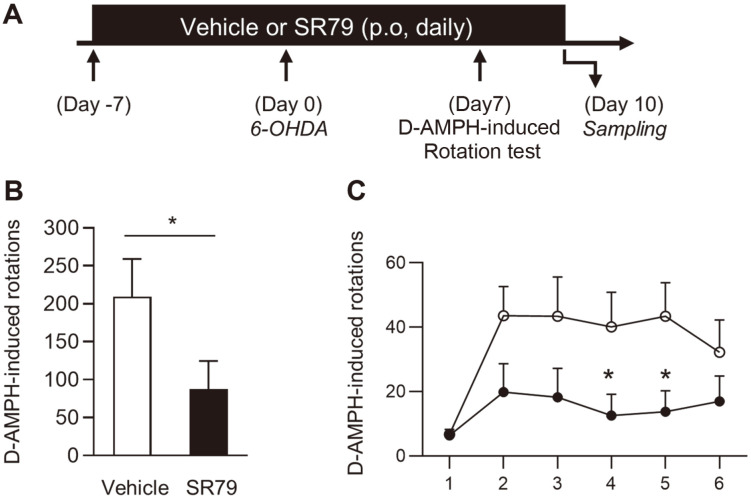
Effect of SR79 on d-AMPH-induced rotations in unilaterally 6-OHDA-lesioned mice. Experimental design for SR79 treatment, 6-OHDA lesion, and behavioral test (**A**). The d-AMPH-induced rotation test was performed seven days after the 6-OHDA lesion (B and C, *n* = 7–8/ group). Time course of rotations counted every 5 min (**B**). Sum of total ipsilateral rotations recorded over 30 min (**C**). Student’s *t*-test. * denotes significant differences between the SR79- and vehicletreated groups at *p* < 0.05. Error bars represent SEM.

The striatum is one of the main inputs of the substantia nigra pas compacta (SNc) and is considered a strategized relay for the expression of motor behaviors [37]. To selectively destroy the nigrostriatal pathway, we targeted the dorsal striatum as the site of 6-OHDA injection in B6 mice. SR79 or vehicle was administered daily by oral gavage for a week and then intrastriatal 6-OHDA lesions were induced in B6 mice (Fig. 1A). On the seventh day of the 6-OHDA lesion, intrastriatal 6-OHDA lesions induced d-AMPH-induced ipsilateral turning behaviors in mice. The d-AMPH-induced ipsilateral rotations were remarkably decreased by SR79 treatment in unilaterally lesioned mice (Figs. 1B and 1C). These results indicated that SR79 improved motor deficits in 6-OHDA-induced PD mice.

### SR79 Increases TH Expression in the Dorsal Striatum of Unilaterally 6-OHDA-Lesioned Mice

**Fig. 2:**
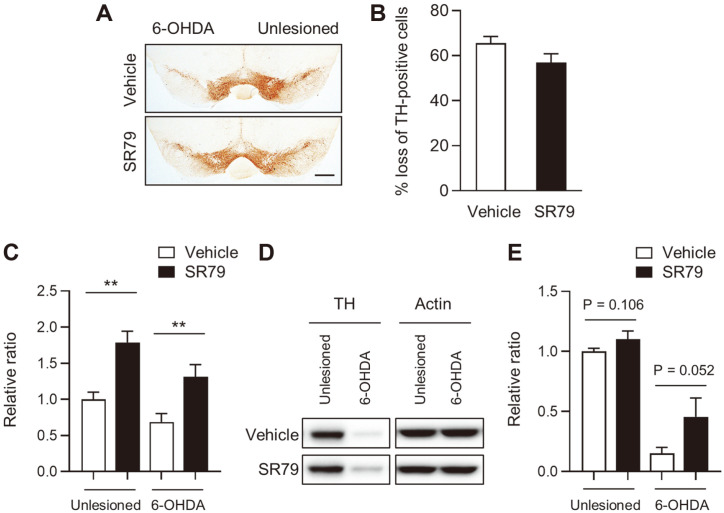
Effects of SR79 on dopaminergic neuron loss in unilaterally 6-OHDA-induced mice. Photomicrograph showing TH-immunoreactive neurons in the SNc (**A**). The scale bar represents 500 μm. The number of TH-positive neurons was measured on the 6-OHDA-lesioned and unlesioned sides of the SNc (**B**). mRNA (**C**) and protein (**D** and **E**) levels of TH were evaluated using western blotting and RT-PCR. TH- and actin-immunoreactivity in the intact and 6-OHDA-lesioned dorsal striatum (**D**). The relative ratio of protein and RNA expression of TH in 6-OHDA-lesioned and unlesioned striata of vehicle-treated and SR79-treated mice (**C** and **E**). Student’s *t*-test. ** denotes significant differences between indicated groups at *p* <0.01. Error bars represent SEM. 6-OHDA, 6-OHDA-lesioned side; unlesioned, intact side; SNc, substantia nigra pars compacta; TH, tyrosine hydroxylase.

The 6-OHDA injection into the dorsal striatum resulted in both the extent of denervation in the dorsal striatum and dopaminergic cell death in the SN [38]. To investigate the neuroprotective effects of SR79 treatment, we performed immunohistochemistry on TH-positive cells. TH catalyzes the conversion of the amino acid L-tyrosine to L-3,4-dihydroxyphenylalanine (L-DOPA) and is used as a marker for dopaminergic cells [39]. We found that 6 μg of 6-OHDA induced dopaminergic cell loss up to 65.60% ± 2.92 in the SNc of mice (Figs. 2A and 2B). SR79 treatment decreased dopaminergic cell death in the SNc (Figs. 2A and 2B, *p* = 0.0532). To determine whether the mRNA and protein levels of TH are regulated by SR79, we performed quantitative RT-PCR and western blotting in the striatum of the mice. Intriguingly, SR79 treatment significantly increased the mRNA expression of Th on both the unlesioned and 6-OHDA-lesioned sides of the dorsal striatum (Fig. 2C, *p* < 0.01). In addition, although it was not significant, TH protein expression was increased by SR79 treatment on both sides of the striatum (Figs. 2D and 2E, *p* = 0.106 and *p* = 0.052). These data suggest that SR79 treatment might regulate Th gene expression in the striatum and suppress dopaminergic cell death in the SNc.

### SR79 Increases the AKT/GSK3β-Mediated Pathway in the 6-OHDA-Lesioned Side of the Dorsal Striatum

**Fig. 3:**
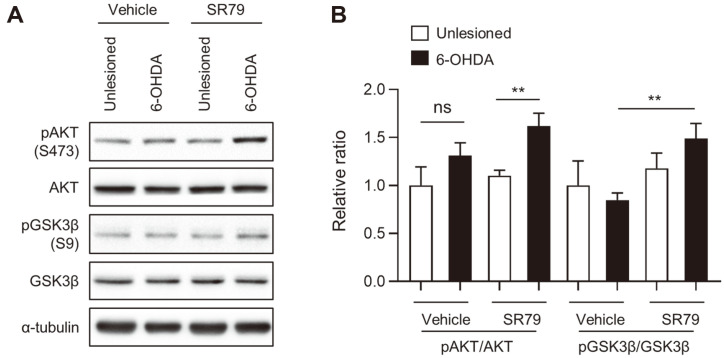
Effects of SR79 on AKT/GSK3β signaling in unilaterally 6-OHDA-lesioned mice. Western blotting using the 6-OHDA-lesioned and intact striata was performed to investigate the protein levels of pAKT, AKT, pGSK3β, and GSK3β (*n* = 6–8/ group). Representative western blot images (**A**). The quantitative value was normalized using α-tubulin and represented relative to the intact side of the striatum in the vehicle-treated group (**B**). Student’s *t*-test. ** denotes significant differences between the indicated groups at *p* <0.01. Error bars represent SEM. Unlesioned, intact side; 6-OHDA, 6-OHDAlesioned side.

Evidence shows that the AKT/GSK3β signaling pathway is associated with the prevention of cellular degeneration [40]. To investigate whether AKT/GSK3β signaling was affected by SR79 treatment, western blot analysis was performed on the unlesioned and 6-OHDA-lesioned sides of the dorsal striatum. Although the phosphorylation of AKT tended to increase on the 6-OHDA-lesioned side compared to that on the intact side (Figs. 3A and 3B), SR79 treatment significantly upregulated pAKT/AKT in the 6-OHDA-lesioned striatum compared to the unlesioned striatum (Figs. 3A and 3B). To determine the effect of SR79 on GSK3β activity, western blotting for pGSK3β at Ser9 was performed. On the ipsilateral side of the striatum, SR79 treatment significantly increased pGSK3β expression compared to the vehicle-treated group (Figs. 3A and 3B, *p* < 0.05). These results indicate that SR79 treatment effectively upregulated the AKT/GSK3β signaling pathway in the 6-OHDA-lesioned side of the striatum.

### SR79 Regulates NRF2-Antioxidative Defense System in Unilaterally 6-OHDA-Lesioned Mice.

**Fig. 4:**
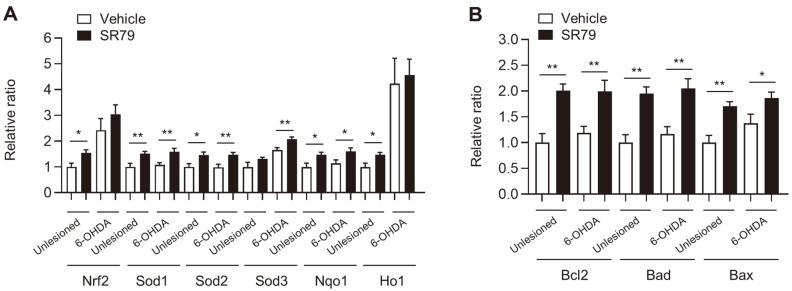
Effects of SR79 on genes involved in oxidative stress and apoptosis in unilaterally 6-OHDA-lesioned mice. RT-PCR using the 6-OHDA-lesioned and intact striatum was performed to explore the mRNA levels of genes involved in oxidative stress (*Nrf2, Sod1, Sod2, Sod3, Nqo1*, and *Ho1*) and apoptosis (*Bcl2, Bad*, and *Bax*) (*n* = 6–8/ group). mRNA levels of the indicated genes are represented as a relative ratio to the intact side of the striatum in the vehicle group (A and B). Student’s *t*-test. * and ** denote significant differences between indicated groups at *p* <0.05 and *p* <0.01. Error bars represent SEM. Unlesioned, intact side; 6-OHDA, 6-OHDA-lesioned side.

One of the major causes of PD is oxidative stress and the accumulation of free radicals, which lead to dopaminergic cell death [41]. Activation of the AKT signaling pathway plays an important role in regulating the Nrf2/antioxidant response element (ARE) pathway and its downstream target genes, including *Sod*, *Nqo1*, and *Ho1* [42]. To determine whether SR79 affects NRF2/ARE signaling, changes in the mRNA expression of related genes were analyzed using RT-PCR. The 6-OHDA lesion significantly increased the expression of *Nrf2*, *Sod3*, and *Ho1* in both vehicle- and SR79-treated groups (Fig. 4A). The mRNA expression of *Nrf2* and *Ho1* was markedly increased by SR79 treatment on the unlesioned side of the striatum but not on the 6-OHDA-lesioned striatum (Fig. 4A). The expression of *Sod1*, *Sod2*, and *Nqo1* was significantly increased by SR79 treatment on both sides of the striatum (Fig. 4A, *p* < 0.05). These data indicate that the administration of SR79 might affect gene expression alterations related to decreased oxidative stress.

### SR79 Increases Bcl-2 Expression in Unilaterally 6-OHDA-Lesioned Mice

It has been reported that the Bcl-2 family plays an important role during apoptosis in response to oxidative stress, which is implicated in PD [43]. We attempted to determine whether SR79 regulated the expression of anti-apoptotic and pro-apoptotic genes, including *Bcl-2*, *Bad*, and *Bax*. Using RT-PCR, we found that the mRNA expression of *Bcl2*, *Bad*, and *Bax* was upregulated by SR79 treatment in both the intact and 6-OHDA-lesioned sides of the dorsal striatum (Fig. 4B, *p* < 0.05 and *p* < 0.01). However, gene upregulation induced by 6-OHDA lesions was not observed on either side of the striatum compared to the unlesioned side. These data indicate that the administration of SR79 might affect the alteration of gene expression related to apoptosis.

### SR79 Attenuates Astrocyte Activation in Unilaterally 6-OHDA-Lesioned Mice

**Fig. 5:**
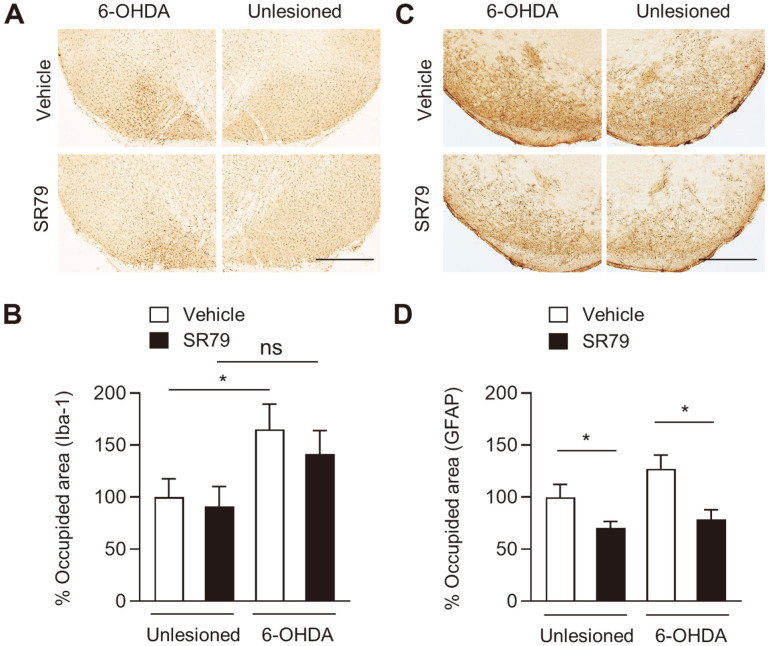
Effects of SR79 on microglial and astrocyte activation in 6-OHDA-lesioned mice. Ten days after 6-OHDA injection, immunohistochemistry for anti-Iba-1 and anti-GFAP antibodies was performed on the brain. Photomicrographs (**A** and **C**) and % occupied area (**B** and **D**) showing Iba-1 or GFAP immunoreactivity in the 6-OHDA-lesioned and unlesioned sides of the SN (*n* = 7–8). Scale bar: 500 μm. * denotes significant difference between the indicated groups at *p* <0.05. ns, not significant.

Previously, we found that SR79 treatment reduces gliosis in the brains of double transgenic mice expressing human amyloid precursor protein and presenilin 1 [23]. In addition, evidence has suggested that gliosis can contribute to the progression of PD neuropathology and that downregulating inflammatory processes can be important to delay the progression of PD [25-28]. To explore the inhibitory effect of SR79 on the activation of microglia and astrocytes, we performed immunohistochemistry for Iba-1 and GFAP in the SN (Figs. 5A-5D). The percentage of the area occupied by Iba-1 and GFAP immunoreactivity was analyzed in the unlesioned and 6-OHDA-lesioned side of the SN. The percentage of area occupied by Iba-1 was increased by 6-OHDA lesions in the vehicle-treated group (Figs. 5A and 5B, *p* < 0.05). In the SR79-treated group, no difference was observed between intact and 6-OHDA lesions. The percentage of the occupied area by immunoreactivity against GFAP significantly decreased by SR79 treatment in both intact and 6-OHDA-lesioned SN (Figs. 5C and 5D, *p* < 0.01), indicating the anti-inflammatory effects of SR79 on 6-OHDA-induced glial activation.

## Discussion

Emerging evidence has shown that gut bacteria can affect immune system regulation and brain health [9, 44-46]. In the present study, we showed that the human gut microbiota SR79 exhibits neuroprotective effects against 6-OHDA-induced neurotoxicity in a mouse model of PD. The effects of SR79 appear to be associated with the regulation of the AKT/GSK3β signaling pathway, induction of gene expression changes, and inhibition of astrocyte activation in the brain.

In this study, the administration of SR79 markedly suppressed asymmetric rotations induced by d-AMPH in unilateral 6-OHDA-lesioned mice. Hemispheric asymmetry is strongly associated with the severity of unilateral lesions [38, 46]. Although not significant, pretreatment with SR79 had an inhibitory effect on dopaminergic cell death. Interestingly, SR79 upregulated TH mRNA and protein expression in both intact and lesioned striata. TH is an important enzyme that synthesizes dopamine by catalyzing the conversion of tyrosine to L-DOPA [39]. Depletion of dopaminergic neurons in the SN results in diminished dopamine synthesis [1, 2]. Investigation of TH regulatory mechanisms, such as gene replacement therapy to increase nigrostriatal TH expression, has been suggested as an effective treatment for PD [48]. The induction of TH expression by SR79 may be beneficial for the pathogenesis of PD. Further studies on the regulation of TH activity and dopamine synthesis by SR79 are required.

Previously, to elucidate the intracellular protective mechanisms leading to SR79 treatment in an AD mouse model, the AKT/GSK3β signaling pathway has been investigated [23]. AKT/GSK3β signaling, a main pathway for neuronal survival [49], was upregulated by chronic administration of SR79 in the mouse model of AD [23]. Activation of AKT signaling decreases GSK3β activity, both in vitro and in vivo [50, 51]. Many cellular functions of GSK3β have been reported, including cell proliferation, cell survival, neuronal plasticity, and neuronal development [52, 53]. Dysfunction of striatal AKT/GSK3β signaling is associated with neuropsychiatric and neurodegenerative disorders [54]. Alterations in striatal AKT/GSK3β signaling have been implicated in toxin-induced neurodegeneration [55, 56]. Moreover, previous studies have suggested that dyskinesia induced by chronic L-DOPA treatment in PD can be ameliorated by the inhibition of GSK3β [32, 57]. To explore the effect of SR79 on GSK3β activity, western blotting for Ser9 was performed. GSK3β is constitutively inactivated by phosphorylation at Ser9 [58]. Although not significant between unlesioned and 6-OHDA-lesioned striata, pGSK3β expression was meaningfully increased in the 6-OHDA-lesioned striatum in the SR79-treated group compared to the vehicle-treated group, indicating the inhibitory effect of SR79 on pGSK3β activity.

A neuropathological feature of PD is the accumulation of free radicals and oxidative stress in the degenerating SN [41]. Evidence has shown that compounds with anti-oxidative properties have neuroprotective effects on PD [59, 60]. The PI3K/AKT signaling pathway plays a critical role in altering the expression of NRF2-regulated genes [61]. An increase in the PI3K/AKT signaling pathway can lead to Nrf2 nuclear translocation and Nrf2-induced expression of Ho-1 and Nqo1, at least in part [62]. The anti-oxidative enzymes such as SOD, HO1, and NQO1, have been suggested to counteract the burden of reactive oxygen species production [42]. Three types of SOD [Cu-Zn SOD (SOD1), Mn-SOD (SOD2), and EC-SOD (SOD3)] catalyze the dismutation of superoxide anions to oxygen molecules and hydrogen peroxide, which are less toxic molecules [63, 64]. In the present study, the gene expression of Sods, Nqo1, and Ho1 was significantly affected by SR79 treatment in the brain. Although further studies are needed to analyze whether these antioxidant enzyme activities are elevated in the presence of SR79, induction of mRNA expression of anti-oxidative enzymes might be an important event in the neuroprotective role of SR79 in PD pathogenesis. In addition, TH-positive neuronal localization of the anti-apoptotic protein Bcl-2 and pro-apoptotic protein Bax has been reported [43]. In an in vitro dopaminergic neuronal cell line, overexpression of Bcl-2 attenuated MPP^+^-induced neuronal death but not 6-OHDA-induced cell death [65]. Increased expression of the anti-apoptotic gene *Bcl2* by SR79 may also be beneficial in 6-OHDA-induced neurotoxicity.

Furthermore, bidirectional communication between the gastrointestinal tract and the central nervous system has been reported [9, 44-46]. Transplants of fecal microbiota reverse neurodegenerative-associated differences in brain immunity [66, 67]. In the present study, to explore the effects of SR79 on neuroinflammation, activation of astrocytes and microglia was assessed by immunohistochemistry for anti-Iba-1 and anti-GFAP antibodies in the SN. At the time of brain preparation, 6-OHDA lesions induced a significant increase in microglial activation but not in astrocyte activation. SR79 treatment significantly inhibits microglial activation in the 6-OHDA-lesioned striatum. Although an increasing trend was observed for astrocyte hyperactivation due to lesions, astrocyte activation was regulated by SR79 in contrast to microglial activation. The area occupied by activated astrocytes was markedly reduced by SR79 treatment on both sides of the striatum. Reactive astrogliosis can secrete inflammatory cytokines and is toxic to healthy neurons [68]. The classical complement cascade components released by reactive astrocytes can enhance synaptic degeneration in neurodegenerative diseases [69]. Reactive astrocytes are present in the brain regions in PD, and neuroinflammation is considered a target for drug discovery in PD [70, 71].

Butyric acid is a short-chain fatty acid produced by gut microbes [72, 73]. Butyric acid has been reported to have epigenetic, anti-inflammatory, and enteroendocrine effects that influences colonic as well as brain function [74]. Decrease in composition of butyric acid-producing bacteria has been observed in the elderly [75]. Fecal SCFAs were markedly reduced in patients with PD compared to individuals of similar age [76]. Several studies have suggested the protective effects of sodium butyrate on rodent models for PD [18, 74, 77]. SR79 is one of the gut commensal species of human in normal condition [22]. The major fermentation end product of glucose metabolism from SR79 is butyric acid [22]. The microbiota-produced butyric acid from prebiotic fermentation could promote both systemic and localized effects [74]. However, further studies are needed to evaluate how the gut commensal species and the microbiota-produced butyric acid affect PD pathophysiology.

In summary, this study provides the first evidence of the neuroprotective effects of SR79 in an animal model of PD. Our data showed that the oral administration of SR79 mitigated d-AMPH-induced rotations and dopaminergic neuronal death in a 6-OHDA-induced rodent model of PD. Although additional studies are needed to validate the anti-parkinsonian effect of SR79 in vitro and also to uncover the underlying molecular mechanisms, the present results suggest that SR79 may be considered a potential therapeutic target for the prevention and treatment of PD.

## Figures and Tables

**Table 1:** Gene names and primer sequences used for RT-PCR analyses.

Gene ID	Gene name	Forward (5'-3')	Reverse (5'-3')
Bcl2	B-cell leukemia/lymphoma 2	TACCGTCGTGACTTCGCAGAG	GCCAGGCTGAGCAGGGTCTT
Bax	Bcl2 associated X protein	CGGCGAATTGGAGATGAACTG	GCAAAGTAGAAGAGGGCAACC
Bad	Bcl2 associated agonist of cell death	GGAAGACGCTAGTGCTACAGA	GAGCCTCCTTTGCCCAAGTTT
Ho1	Heme oxygenase 1	CAGCCCCACCAAGTTCAAAC	GGCGGTCTTAGCCTCTTCTGT
Nqo1	NAD(P)H quinone dehydrogenase 1	GGTTTACAGCATTGGCCACACT	AACAGGCTGCTTGGAGCAAA
Nrf2	Nuclear factor erythroid 2-related factor 2	GGCCTTTTTTGCTCAGTTTCA	ATGTGGGCAACCTGGGAGTA
Sod1	Copper-zinc superoxide dismutase	GCGGTGAACCAGTTGTGTTG	CCCATACTGATGGACGTGGAA
Sod2	Manganese superoxide dismutase	CCACACATTAACGCGCAGAT	TCGGTGGCGTTGAGATTGT
Sod3	Extracellular superoxide dismutase	GCGGCCTGTGGCTCTGTCA	GCGTGTCGCCTATCTTCTCAA
TH	Tyrosine hydroxylase	CCGTCATGCCTCCTCACCTA	CGGTCAGCCAACATGGGTAC
18S	18S ribosomal RNA	GACACGGACAGGATTGACAGATTGATAG	GTTAGCATGCCAGAGTCTCGTTCGTT
